# Moving Forward with Medical Education in Times of a Pandemic: Universities in Romania Double Down on Virtual and Blended Learning

**DOI:** 10.25122/jml-2020-1008

**Published:** 2020

**Authors:** Dafin Muresanu, Anca Dana Buzoianu

**Affiliations:** “Iuliu Hatieganu” University of Medicine and Pharmacy, Cluj-Napoca, Romania

Education is one of the primary pillars of building healthier societies. In a world that has become more and more digitized, education should adapt to the public’s needs, coming as a gap-bridging activity in the quest for knowledge and personal growth. In trying times, the pursuit of education represents the hope of society. The world still faces the challenges of polarization, discrimination and inequity. These unfortunate realities create an ever-growing need for connection, innovation and societal progress. In areas where heterogeneity makes for the norm, new forms of learning could bridge disparities and allow people to develop to their full potential. With minimal resources and maximized access, e-learning accounts for the next step in building innovation-driven societies, designing new tools for connection, and aiding students and teachers alike to achieve their full potential.

The Covid-19 pandemic has put a never-before-seen strain on the educational system [1]. There is a growing need to fundamentally restructure the educational process to be safe and effective for both students and teachers. The pandemic has highlighted and deepened disparities and increased heterogeneity around the globe, in education and various other sectors [2]. However, the public health crisis has become a catalyst for innovation and support in education and training continuity [2]. In challenging times, human creativity and shared efforts represent valuable tools to create new resources, new ideas, and new perspectives. We are looking into a possible reconceptualization of the educational process itself, one that could better fulfill the needs and wishes of the new generations [3].

Nevertheless, in the quest of finding new applications and new perspectives on the digitalization of education, all efforts need to be evaluated, finely tuned, and developed with concern for long-term sustainability. In the quest to implement new and exciting ideas, one should be wary of the limitations of new technology and bear in mind the impact this transition holds on students’ and teachers’ mental health [4, 5]. With all limitations considered, e-learning still makes for an exciting opportunity, supporting more flexibility in the development and delivery of the study programs, making for an exciting approach to education even outside the pandemic context.

There are various resources that could be implemented in the context of online and blended learning. A first resource would be Massive Open Online Courses (MOOCs) [6]. This is one tool for recognizing institutional e-learning, creating the opportunity for credit transfer, encouraging students to follow interests, and personalize their learning experience while creating a more flexible learning experience.

Moreover, it helps track ongoing progress and identify learning strengths and weaknesses [7]. Chatbots, a conversational intelligent tutoring system (CITS), and game-based learning could also make useful tools, standing for an accessible pathway to information, improvement of the learning experience [8-10]. It is, without doubt, a direction worthy of consideration. In the context of e-learning, the technology works together with educational sciences, psychology, and design to create a wholesome personalized experience for the most “tech-savvy” generations of students. Although subject to skepticism, the concept of digital education could be the future of the educational process.

Assessment remains a significant issue, considering the rapid and unexpected switch to online, and, due to that, adaptation for those most impacted should be considered. Replacing exams with coursework or online assessment, removing rigid time constraints, extending the registration period, and adapting the marking system are vital steps to ensure a fair evaluation, control teacher bias, and ease students’ burden [11]. In e-learning assessment, creating mitigating circumstances policies to cover the students’ diverse needs, in the context of accessibility, transparency, consistency, and the ability to be manageable for staff is vital. In these times, consideration should be given to students facing debilitating circumstances (anxiety disorders, students with dependent children) [11]. With digitized education, some challenges may arise concerning network infrastructure and Information and Communication Technology (ICT) knowledge, especially in developing countries [12]. However, e-learning has excellent potential for meeting the growing educational demand in developing countries, considering the shortage of expert teachers, updated textbooks, and teaching materials [12]. It furthermore opens the door for virtual mobility for students who would, otherwise, due to social, financial, or various other reasons, would not have access to this type of educational exposure [13, 14].

Virtual mobility provides students with access to an international experience online and can be implemented by itself or combined with a physical mobility scheme [14, 15]. These programs often include virtual student meetings, access to the university’s library, and student support [14]. The mobilities address some limitations of traditional university exchanges and allow students to visit several universities from their home’s comfort to build a more specialized and individualized study program [14, 15]. One great benefit of online education is represented by its cost-effectiveness, which is applied both in the case of online education and the blended model [16]. Due to the use of multimedia, lack of spatial and temporal constraints, higher admission rates, easy and facile access to information, digitization manages to reduce costs while also addressing the issue of human resources [16].

Blended learning (a combination of online teaching and learning with face-to-face interaction) is one valuable innovation embraced in the teaching and learning environment [17]. Although challenging in some aspects (user interaction with technology, commitment), it makes for a great opportunity of expanding the limits of education [17]. Factors such as age, self-regulation, computer confidence, family and social support, attitude towards blended learning, and technology might impact the implementation and success of blended learning [17]. However, none of the mentioned factors are a significant predictor of performance [17]. Another advantage of blended learning would be a better balance between work and study, as it eases the study workload management [17]. This method’s potential for effectiveness is highlighted by plenty of reviews [3, 17, 18], considering increased access rates for various study cohorts, improved success rates, and preference of the students [12]. It is a source of great opportunities and challenges, as it interacts with most aspects of higher education [12], breaking assumptions and creating a new approach to learning.

In Romania, the “Iuliu Hatieganu” University of Medicine and Pharmacy from Cluj-Napoca (UMF Cluj) has made significant strides in adapting its educational programs to our current reality. In partnership with the RoNeuro Institute for Neurological Research and Diagnostic, UMF Cluj is now spearheading an effort to deliver landmark keynote presentations from its International Doctoral School of Neuroscience in a digital format, freely available for students and staff. Other institutions that have made strenuous progress into joining the digitization movement, such as the “Carol Davila” University of Medicine and Pharmacy from Bucharest, are also joining this project, which partners hope to develop into a prospective endeavor as well.

In 2021, UMF Cluj will also join forces with partners from the NeurotechEU Alliance – the European University of Brain and Technology (www.theneurotech.eu), making an effort to build a trans-European network of excellence in brain research and technologies to increase the competitiveness of European education, research, economy and society. It is our firm belief that universities worldwide can work together through the digitization process to ensure high quality and innovative approaches, expanding the array of available resources and expertise and bridging connections among researchers, educators, and students.

Virtual and blended learning will define the following decades in the evolution and revolution of education. Change can be fearsome, as there are many contingencies to be considered, such as the influence of digitization on human interaction - technology should promote human interaction, rather than standing in as a substitute. Opportunities brought about by e-learning are exciting and inspiring, and frankly, our only chance of moving forward. Through creating connections and bridging existing gaps, e-learning could be the cornerstone in the future pursuit of knowledge.
